# Reply to: Validity of ultrasound in predicting acute appendicitis among children, keeping histopathology as gold standard: Methodological issues

**DOI:** 10.1016/j.amsu.2019.06.016

**Published:** 2019-07-13

**Authors:** Ubaidullah khan

**Affiliations:** Pediatric Surgery, Department of Surgery, Alhada Armed Forces Hospital, Taif, Saudi Arabia

**Keywords:** Ultrasound, Histopathology

Thank you for this interest in our paper, we hope this reply will be helpful in regard to your valuable comments [1].

The aim of our study was to determine the accuracy of ultrasound in diagnosis of acute appendicitis in children keeping histopathology as gold standard [1]. A few limitations of our study are worth mentioning, first we could not capture all the population going through ultrasound for that given period, due to patients seen by emergency staff, missing negative predictive value (NPV) in our study. Likewise, the non-operative patient, no histopathology, out of domain and not co-relate to title aim, Tristan.R et al. has good visualization but a limitation of their study was the lack of pathological evidence in children that did not undergo appendectomy [2]. Lastly the study was limited to single center. Therefore, methodological issues taken into account, there was no impact on our outcome, as its with ultrasound verses histopathology.

We strongly agree that the most appropriate estimates to evaluate validity of a single test such as sensitivity (Sen), specificity (Spe), positive predictive value (PPV), negative predictive value (NPV). Therefore, measuring predictive values was essential and we could not collect all populations during time period of our study. Considering NPV was a great point raised and will be matter of consideration in future research work.

Yes we agree on the point of using ultrasound for diagnosis and as well as to declear normal appendix, but, reason was not the aim of our study.

As has been stated, our aim was ultrasound verses histopathology, the design of study was accurate as well collection of data. However, as mentioned in your letter, different aspect can be study in this regard to be more specific and accurate, but, we choose simple method to raised the issue in aiding diagnosis of acute appendicitis and lessen the negative appendectomy rate. Our study showed promising results in this regard, and in future further studies needed to more widen the scope of accuracy in diagnosis of appendicitis. These can be achieved by multi-center cohart studies and keeping the fact regarding PPV, NPV, etc.

With due respect to your conclusion remarks, its cross sectional study, the measures taken into account was well planed and according to the design choosen. In our study we cross link with histopathology which is more accurate method in regard to decrease negative appendectomy rate, as ultrasound is dependent on operater, which leed you further in mis-interpretion of results, as mention by P. Gerbier et al. prompt radiological-surgical correlation could help to improve resident performance [3]. We minimize this issue very well, further, we choose patient which went for surgery, to look deepen into facts finding and promising outcome, such misleading results which you mention could not be seen.

Therefore, we believe that our study was initiated as an example to open the chapter for further wider broad comprehensive research work such as its prevalence, severity, valdility and predictivity. Many methods and designs option, likewise, the one we choose and proof the aim in a way to bring accuracy of diagnosis to high percentage. Similary if you read paper from BS Kelly et al. exact aim and protocol choosen as ours, they cross ultrasound verse histopathology. Important to highlight in respond to your letter, are co-related and their final outcome not more promising then as ours, false positive (FPs) n = 20 and false negative (FNs) n = 28 [4]. Hence, in the end you will agree that our study was rightly chosen for the purposed aim and methodology.

## Provenance and peer review

Authors invited to reply, internally peer reviewed.

## Ethical approval

Hospital ethical comity approve the study in our hospital.

## Sources of funding

Personal.

## Author contribution

Ubaidullah khan:

## Conflicts of interest

No interest.

## Research registry number

Following is the number.

researchregistry3713.

## Guarantor

DR ubaidullah khan.
